# Complete Genome Sequences of a Clinical Isolate and an Environmental Isolate of Vibrio parahaemolyticus

**DOI:** 10.1128/genomeA.00216-15

**Published:** 2015-03-26

**Authors:** Catharina H. M. Lüdeke, Nguyet Kong, Bart C. Weimer, Markus Fischer, Jessica L. Jones

**Affiliations:** a Gulf Coast Seafood Laboratory, Division of Seafood Science and Technology, Food and Drug Administration, Dauphin Island, Alabama, USA; b Hamburg School of Food Science, University of Hamburg, Hamburg, Germany; c School of Veterinary Medicine, University of California, Davis, California, USA; d 100K Pathogen Genome Project, University of California, Davis, California, USA

## Abstract

Vibrio parahaemolyticus is the leading cause of seafood-borne infections in the United States. We report complete genome sequences for two V. parahaemolyticus strains isolated in 2007, CDC_K4557 and FDA_R31 of clinical and oyster origin, respectively. These two sequences might assist in the investigation of differential virulence of this organism.

## GENOME ANNOUNCEMENT

Vibrio parahaemolyticus is a halophilic Gram-negative bacterium naturally occurring in estuarine environments (1). Through consumption of raw or undercooked seafood, or contact with contaminated seawater, V. parahaemolyticus can cause infections in humans; gastroenteritis is typical, but rarely, wound and/or sepsis infections occur. V. parahaemolyticus is the leading cause of seafood-borne gastroenteritis in the United States (2), and there has been an increase in reported illnesses in the last two decades (3).

V. parahaemolyticus can carry the thermostable direct hemolysin (*tdh*) and *tdh*-related hemolysin (*trh*) genes, which are generally associated with pathogenicity and are used in outbreak investigations and assessing risk (4, 5). Clinical isolates more frequently carry the *tdh* and/or *trh* genes than environmental isolates (6). However, in recent studies, clinical isolates lacking both of these genes have been identified (7). Tissue culture studies have revealed that the presence of *tdh* had no effect on cytotoxicity (8, 9). Hence, additional virulence factors likely exist for V. parahaemolyticus.

We sequenced two V. parahaemolyticus isolates, CDC_K4557 and FDA_R31, to better understand the pathogenic potential of these isolates and eventually improve risk assessment. CDC_K4557 was isolated from the stool of a patient in Louisiana in 2007 and submitted to the Centers for Disease Control and Prevention (CDC). FDA_R31 was isolated by the Food and Drug Administration (FDA) from an oyster sample harvested in Louisiana in 2007. As the clinical isolate is *tdh*^−^ *trh*^−^ and the oyster isolate is *tdh*^+^ *trh*^+^ by PCR, these strains are ideal for identifying new and/or additional virulence markers.

The genomes were sequenced within the University of California at Davis 100K Pathogen Genome Project using the PacBio RSII sequencing platform (Pacific Biomarkers, Menlo Park, CA, USA). High-molecular-weight gDNA was extracted from overnight cultures grown on Trypticase soy agar, lysed with an enzyme cocktail, purified with the QIAamp DNA minikit (Qiagen, Valencia, CA, USA), and analyzed on a 2200 TapeStation system with the Genomic DNA ScreenTape (Agilent Technologies, Santa Clara, CA, USA) assay for integrity of high molecular weight gDNA (10). After evaluation of gDNA size and quantity, 10 µg was used for fragmentation using the Covaris g-TUBE device (Covaris, Woburn, MA, USA) following the manufacturer’s instructions (11). The fragmented gDNA was used for library construction with the PacBio SMRTbell 10kb Library preparation kit, which was normalized to 1 to 5 µg input. Libraries were sequenced utilizing PacBio RSII and C2 chemistry with 100× coverage per the manufacturer’s instructions. For each isolate, the genomic sequence single-pass reads were *de novo* assembled using the Hierarchical Genome Assembly Process (HGAP) version 1.4 software (Pacific Biosciences) and were then annotated using the NCBI Prokaryotic Genomes Automatic Annotation Pipeline (http://www.ncbi.nlm.nih.gov/genome/annotation_prok) (12). Through the annotation process, 4,771 and 4,937 genes for the clinical and oyster isolates, respectively, as well as 4,579 and 4,731 coding regions were identified. The presence or absence of the *tdh* and *trh* genes was confirmed in both isolates.

### Nucleotide sequence accession numbers.

The closed genome sequences of the two V. parahaemolyticus isolates are available in GenBank under the accession numbers CP006004 and CP006005 for chromosomes I and II of FDA_R31, respectively, and CP006008 and CP006007 for CDC_K4557. The versions described in this paper are the first versions.
